# A Collaborative Learning Model for Skin Lesion Segmentation and Classification

**DOI:** 10.3390/diagnostics13050912

**Published:** 2023-02-28

**Authors:** Ying Wang, Jie Su, Qiuyu Xu, Yixin Zhong

**Affiliations:** 1 School of Information Science and Engineering, University of Jinan, Jinan 250022, China; 2 Shandong Provincial Key Laboratory of Network Based Intelligent Computing, University of Jinan, Jinan 250022, China; 3 Artificial Intelligence Research Institute, University of Jinan, Jinan 250022, China

**Keywords:** skin cancer, segmentation, classification, self-training, class activation mapping

## Abstract

The automatic segmentation and classification of skin lesions are two essential tasks in computer-aided skin cancer diagnosis. Segmentation aims to detect the location and boundary of the skin lesion area, while classification is used to evaluate the type of skin lesion. The location and contour information of lesions provided by segmentation is essential for the classification of skin lesions, while the skin disease classification helps generate target localization maps to assist the segmentation task. Although the segmentation and classification are studied independently in most cases, we find meaningful information can be explored using the correlation of dermatological segmentation and classification tasks, especially when the sample data are insufficient. In this paper, we propose a collaborative learning deep convolutional neural networks (CL-DCNN) model based on the teacher–student learning method for dermatological segmentation and classification. To generate high-quality pseudo-labels, we provide a self-training method. The segmentation network is selectively retrained through classification network screening pseudo-labels. Specially, we obtain high-quality pseudo-labels for the segmentation network by providing a reliability measure method. We also employ class activation maps to improve the location ability of the segmentation network. Furthermore, we provide the lesion contour information by using the lesion segmentation masks to improve the recognition ability of the classification network. Experiments are carried on the ISIC 2017 and ISIC Archive datasets. The CL-DCNN model achieved a Jaccard of 79.1% on the skin lesion segmentation task and an average AUC of 93.7% on the skin disease classification task, which is superior to the advanced skin lesion segmentation methods and classification methods.

## 1. Introduction

Skin cancer is one of the most common and deadly cancers. The American Cancer Society reports that by 2022, there will be approximately 97,920 new cases of melanoma [1]. The early diagnosis and treatment of skin cancer are critical. Except for early surgical excision, skin cancer lacks special treatment and has a poor prognosis. Therefore, the computer-aided diagnosis of skin diseases has been increasingly investigated to assist dermatologists in improving diagnosis accuracy, efficiency, and objectivity.

Accurate detection of the skin lesion’s boundary can help pathologists mitigate noise interference and obtain contour information [2]. With a large amount of labeled data, deep learning has achieved advanced performance in image processing. However, obtaining pixel-level annotations for segmentation is often expensive for dermoscopic images, as generating accurate annotations requires specialized skills [3]. Many semi-supervised learning and weakly supervised learning methods have been proposed for segmentation in the case of small quantity of pixel-level labeled data. These methods use unlabeled or weakly labeled data to realize accurate segmentation. Self-training is a semi-supervised method that uses a teacher model, trained using labeled data, to create synthetic labels for unlabeled examples [4]. The student model can be trained with the pseudo-labels generated by the teacher model [5]. Weakly supervised learning is an umbrella covering a variety of studies that attempt to construct predictive models by learning with weak supervision [6]. In the weakly supervised learning method, the image-level labeled data can be used to train the classification networks to generate class activation maps (CAMs) [7]. Then, the pseudo-labels generated by CAMs are employed to train the segmentation network to improve the segmentation performance.

The classification of skin diseases (melanoma, nevus, seborrheic keratosis) is essential to assist physicians in diagnosing skin cancer. The dermatological classification task is challenging due to four reasons: (1) the low contrast between each lesion and its surrounding skin tissue results in fuzzy lesion boundaries; (2) the inter-type skin lesions may share visual similarities, and the intra-type lesions may have visual differences; (3) skin lesions vary significantly in visual appearance, which may be corrupted by artifacts such as hair, blood vessels, and air bubbles; and (4) labeling skin disease types on dermatoscopic images requires specialized knowledge, resulting in a small amount of image-level labeled training data. The segmentation can help remove distractions from dermoscopic images and thus is highly beneficial for improving the accuracy of lesion classification [2]. For many medical image classification methods, accurate segmentation is considered as the first step of the classification task. Many researchers [8,9,10,11] focus on the methods based on mask to improve the classification performance. The different scenarios included approaches that exploited the segmentation masks either for the cropping of skin lesion images or removing the surrounding background or using the segmentation masks as an additional input channel for model training [11]. However, inaccurate masks may disturb the judgment of the classification network. When using masks to improve classification performance, the importance of mask accuracy should be emphasized.

**Figure 1:**
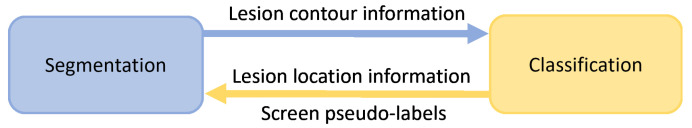
The correlation between skin lesion segmentation and classification tasks. Segmentation can provide the contour information of lesions for classification. Classification can generate class activation maps to provide the location information of lesions for segmentation. Classification also can be used to screen pseudo-labels for segmentation.

In most cases, the segmentation and classification of skin lesions are studied independently. As Figure 1 shows, to improve the skin lesion segmentation and classification performance under limited annotation data, we explore the correlation between segmentation and classification tasks to enable segmentation and classification to learn more helpful information.

This project proposes a deep convolutional neural network model, termed CL-DCNN, for the collaborative learning of dermatological classification and segmentation. The contributions of this work are three-fold:

(1) We propose a CL-DCNN model for accurate skin lesion segmentation and classification. Different from the methods dedicated to segmentation or classification, the model tries to leverage the intrinsic correlation in segmentation and classification tasks, improving segmentation and classification performance with limited annotation data.
(2) We provide a self-training method for segmentation by generating high-quality pseudo-labels. Specifically, to alleviate the potential segmentation performance degradation incurred by incorrect pseudo-labels, we screen reliable pseudo-labels based on the similarity between pseudo-labels and ground truth for selective retraining.
(3) We employ class activation maps to improve the location ability of the segmentation and apply lesion masks to improve the recognition ability of the classification.

## 2. Related Work

### 2.1. Segmentation and Classification of Skin Lesion

In medical image processing, automatic disease diagnosis has been widely explored and applied to various practical computer-aided diagnosis and treatment systems [12]. Classification and segmentation are two fundamental tasks in dermatoscopy image processing. Classification can predict the type or severity of skin disease, and segmentation aims to identify pixel-level fine-grained lesion regions.

The lesion area’s shape information is essential for skin disease discrimination. Existing works have explored the method of skin lesion segmentation to assist dermatologists in diagnosing diseases. Lei et al. [13] proposed a generative adversarial network that enhances the decision making of the discriminative module through joint learning. Wang et al. [14] introduced a new knowledge-aware depth framework to integrate clinical knowledge into the task of skin lesion segmentation. Wang et al. [15] integrated a novel boundary attention gate into the transformer, enabling the network to model global long-range dependencies and capture more local details. Bi et al. [16] fused the extracted user input and image features in multiple stages to alleviate the information loss. Mirikharaji et al. [17] encoded the star shape prior to the loss function, which penalizes non-star shape segments in FCN prediction maps to guarantee a global structure in segmentation results. Wang et al. [18] designed a novel bi-directional dermoscopic feature learning framework, which models the complex correlation between skin lesions and their informative context.

Automatic skin lesion classification in dermoscopic images is critical to improving diagnostic accuracy and reducing melanoma mortality [19]. Li et al. [20] proposed a difficulty-aware meta-optimization scheme to address the classification of rare diseases, which is optimized by dynamically down-weighting easy tasks and emphasizing complex tasks. Yu et al. [21] used sequential dermoscopic images for early melanoma diagnosis, reducing the misdiagnosis of borderline cases caused by lesions’ temporal and morphological changes. Zhang et al. [19] designed an attention residual learning block that jointly uses residual learning and novel attention learning mechanisms to improve the classification network’s ability for discriminative representation. Zhang et al. [22] simultaneously used dual DCNNs with the asynergic network, which can mutually learn from each other to address the challenges caused by the intra-class variation and inter-class similarity in skin lesion classification.

### 2.2. Segmentation and Classification Collaborative Learning

Segmentation can provide the location and contour information of the skin lesion for classification. The benefits of segmentation to classification motivate researchers to solve problems through collaborative learning of multiple tasks [10]. Yu et al. [8] designed classification networks to use segmentation results to learn more representative and specific features, alleviating the shortage of training data. Shen et al. [9] proposed a mixed-supervision guided method and a residual-aided classification U-Net model for joint segmentation and benign–malignant classification. Xie et al. [10] used multi-task generative adversarial networks to generate accurate masks to improve classification performance. Mahbod et al. [11] studied the effect of using segmentation masks in different ways on the performance of dermatological classification.

The potential benefit of classification results to the lesion segmentation task can be achieved using the weakly supervised learning strategy [23]. This method is usually implemented by CAMs [7] to locate objects of interest in images to train the segmentation network. Zhang et al. [24] leveraged an image classification branch to generate CAMs for the annotated categories, which are further pruned into confident yet tiny object/background regions. Jo et al. [25] proposed the Puzzle-CAM algorithm to narrow the supervision gap between fully supervised semantic segmentation and weakly supervised semantic segmentation using image-level labels. Wei et al. [26] used classifiers to activate hard-to-discriminate regions to improve segmentation performance. Qin et al. [27] designed the spotlight branch and compensation branch to obtain weighted CAMs to provide supervisory signals for recalibration. Yuan et al. [28] reported a gated recurrent network with dual classification assistance for semantic segmentation to solve the blurred boundaries problem.

Many methods use the potential correlation between segmentation and classification tasks, which are tasks that can learn from each other. Zhou et al. [12] jointly improved the performance of disease grading and lesion segmentation through a semi-supervised collaborative learning method with an attention mechanism. Xie et al. [23] proposed a mutual bootstrapping model for automated skin lesion segmentation and classification. Jin et al. [29] designed a cascaded knowledge diffusion network to transfer and aggregate the knowledge learned from different tasks.

### 2.3. Self-Training for Segmentation

To fully use the unlabeled data to improve the segmentation performance, Yang et al. [5] performed selective retraining by ranking the reliability of unlabeled images based on overall prediction-level stability. Wang et al. [30] separate reliable and unreliable pixels via the entropy of predictions, push each unreliable pixel to a category-wise queue consisting of negative samples, and train the segmentation model with all candidate pixels. Zheng et al. [31] explicitly estimate the segment prediction uncertainty with the assistance of an auxiliary classifier and then ignore the unreliable pixel while self-training to improve the segmentation performance.

Despite the impressive results obtained by the above methods, they do not pay enough attention to the correlation between segmentation and classification tasks. Therefore, we design a CL-DCNN model based on the relationship between tasks. The correlation between classification and segmentation is paid more attention to by filtering reliable pseudo-labels, generating masks, and generating class activation maps. The classification and segmentation task can collaboratively learn more information under limited labeled data.

## 3. Method

### 3.1. Problem Definition

**Figure 2:**
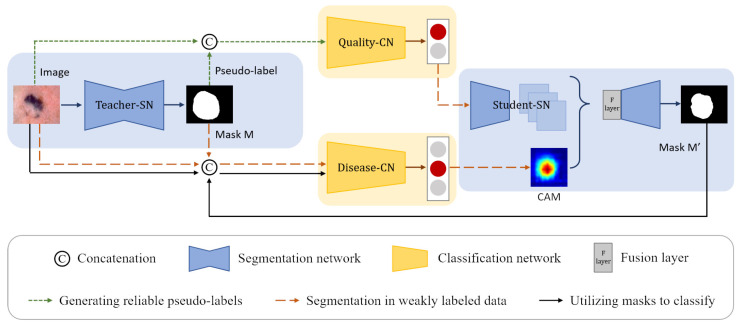
Structure of the CL-DCNN model for skin lesion segmentation and classification. The teacher-SN is constructed to generate the pseudo-labels, which are concatenated with original images as the input to train the quality-CN to screen reliable pseudo-labels for self-training. Then, the masks generated by teacher-SN are employed to provide disease-CN with information about the lesions and promote disease-CN, which can generate accurate CAMs. Following that, we take advantage of CAMs and reliable pseudo-labels to improve the student-SN’s skin lesion segmentation performance. In the end, the masks generated by student-SN are employed to improve the disease-CN’s skin disease identification ability.

We propose a CL-DCNN model for accurate dermatological segmentation and classification, which consists of four networks: the teacher segmentation network, the pseudo-label quality evaluation network, the skin disease classification network, and the student segmentation network. In this model, some terms and definitions are shown in Table 1, and the pipeline is summarized in Figure 2.

### 3.2. Generating Reliable Pseudo-Labels

In the self-training scheme [32], unlabeled data can be used to generate pseudo-labels to help the segmentation network to learn more about the image under limited labeled data. However, some pseudo-labels generated by trained teacher segmentation network are of poor quality. If pseudo-labels with uneven quality are directly employed to train the student segmentation network, it is accessible to overfit the noise. We hope the CL-DCNN model can automatically obtain reliable pseudo-labels. To realize this task, we need to solve three problems: (1) how to generate pseudo-labels, (2) how to screen reliable pseudo-labels, and (3) how to obtain reliable pseudo-labels. Therefore, we design a reliable pseudo-labels generate method based on the similarity between pseudo-labels and ground truth for selective retraining. This method can realize the automatic screen of reliable pseudo-labels by training a pseudo-label quality evaluation classification network.

#### 3.2.1. Generating Pseudo-Labels

**Figure 3:**
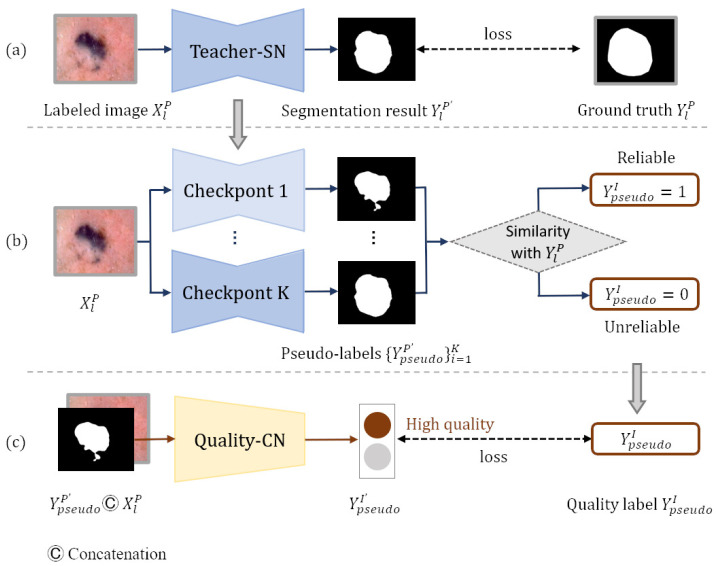
Generating reliable pseudo-labels: (**a**) generating pseudo-labels, (**b**) generating pseudo-label quality level training set, and (**c**) screening reliable pseudo-labels.

In order to generate pseudo-labels, we build a teacher segmentation network teacher-SN based on Deeplabv3+ [33], which is pre-trained on the MS-COCO [34] and PASCAL VOC 2012 [35] datasets. To adapt the Deeplabv3+ network to the skin lesion segmentation task, we remove its last convolutional layer and then add a new convolutional layer with the output channel of one for prediction. The weights of the new layer are randomly initialized, and the activation function in the last layer is set to the sigmoid function. Pixels at the edge of lesions are usually difficult to classify, so we employ rank loss [23] to promote the segmentation network to focus on hard pixels and learn more discriminative representations. Figure 3a shows that the teacher-SN is trained with the segmentation training set $D_{l}^{P}$. The pseudo-labels can be produced by inputting the unlabeled images into the trained teacher-SN. However, the incorrect predictions in some hard examples may negatively impact the following self-training process.

#### 3.2.2. Screening Reliable Pseudo-Labels

To realize the automatic screen of reliable pseudo-labels, we build a pseudo-label quality evaluation network quality-CN and then generate an classification training set that presents the quality grade of pseudo-labels for the training of quality-CN.

The quality-CN is built upon the advanced Xception network [36], which is pre-trained on the ImageNet dataset [37]. After performing global average pooling, features are input to a fully connected layer of *C* randomly initialized neurons followed by a softmax activation function. The quality-CN aims to classify the reliability of pseudo-labels (reliable or unreliable) after inputting the original images and pseudo-labels. Therefore, the *C* is set as 2. We optimize quality-CN by minimizing the cross-entropy loss.

Inspired by ST++ [5], we generate an image-level pseudo-label quality grade training set $D_{pseudo}^{I}$ based on the prediction level in the entire training course for the training of quality-CN. $D_{pseudo}^{I}$ consists of kN dermatology images ${\left\{X_{l}^{P}\right\}}_{i=1}^{kN}$, pseudo-labels ${\left\{Y_{pseudo}^{P^{\prime }}\right\}}_{i=1}^{kN}$, and the image-level quality labels ${\left\{Y_{pseudo}^{I}\right\}}_{i=1}^{kN}$ of pseudo-labels. Pseudo-label $Y_{pseudo}^{P^{\prime }}$ is generated by inputting each dermatology image $X_{l}^{P}$ from dataset $D_{l}^{P}$ into teacher-SN’s checkpoint. The quality label $Y_{pseudo}^{I}$ presents the reliability of the pseudo-label, which is obtained by calculating the similarity with ground truth $Y_{l}^{P}$ ($Y_{l}^{P}\in D_{l}^{P}$). The plainest process of generating the pseudo-label quality grade training set $D_{pseudo}^{I}$ is shown in Figure 3b. Since the training model tends to converge and achieves different performances in the middle training stage, we input each image $X_{l}^{P}$ from dataset $D_{l}^{P}$ into *k* checkpoints of the teacher-SN to generate *k* pseudo-labels ${\left\{Y_{pseudo}^{P^{\prime }}\right\}}_{i=1}^{k}$ of different qualities. Checkpoints are intermediate models that have not fully converged, and they are often used to save parameters. Then, to measure the reliability of each pseudo-label $Y_{pseudo}^{P^{\prime }}$, we compute the Jaccard score *s* between pseudo-label $Y_{pseudo}^{P^{\prime }}$ and ground truth $Y_{l}^{P}$:

(1) $$s=\mathrm{Jaccard}\left(Y_{l}^{P},Y_{pseudo}^{P^{\prime }}\right)=|Y_{l}^{P}\cap Y_{pseudo}^{P^{\prime }}\left|/\right|Y_{l}^{P}\cup Y_{pseudo}^{P^{\prime }}|$$

The Jaccard can serve as a measurement for stability and further reflect the reliability of the $Y_{pseudo}^{P^{\prime }}$. Based on the Jaccard scores, the pseudo-labels are classified into high-quality ($Y_{pseudo}^{I}=1$) and low-quality ($Y_{pseudo}^{I}=0$).

(2) $$Y_{pseudo}^{I}=\{\begin{array}{cc} 1 & s\geq t\\ 0 & s<t\; \end{array}$$

*t* is a threshold that is obtained by empirical. $D_{pseudo}^{I}$ is generated based on the quality grade of the pseudo-labels.

After obtaining the classification training set $D_{pseudo}^{I}$, the quality-CN is trained to evaluate the quality of the pseudo labels. As shown in Figure 3c, each image $X_{l}^{P}$ and corresponding pseudo-label $Y_{pseudo}^{P^{\prime }}$ are concatenated along the dimension of the channel and input into quality-CN. The quality-CN is trained according to the category label $Y_{pseudo}^{I}$ in $D_{pseudo}^{I}$.

#### 3.2.3. Obtaining Reliable Pseudo-Labels

$D_{u}$ is an unlabeled segmentation training set containing *n* pieces of unlabeled images ${\left\{X_{u}\right\}}_{i=1}^{n}$. We input each unlabeled image $X_{u}$ from dataset $D_{u}$ into teacher-SN to generate pseudo-label $Y_{u}^{P^{\prime }}$. After that, the *n* pieces of unlabeled images ${\left\{X_{u}\right\}}_{i=1}^{n}$ with their corresponding pseudo-labels ${\left\{Y_{u}^{P^{\prime }}\right\}}_{i=1}^{n}$ are concatenated along the dimensionality of channels and then input to the trained quality-CN to screen $n^{\prime }$ $\left(n^{\prime }<n\right)$ reliable pseudo-labels $Y_{u}^{P^{\prime \prime }}$. The screened reliable pseudo-label dataset is denoted by $D_{\mathrm{pseudo}}^{P}={\left\{\left(X_{u},Y_{u}^{P^{\prime \prime }}\right)\right\}}_{i=1}^{n^{\prime }}$. The pseudocode of generating reliable pseudo-labels is illustrated in Algorithm 1, which works as a strong baseline for our self-training method.

### 3.3. Segmentation in Weakly Labeled Data

In addition to unlabeled data, image-level labeled data also can be used to train segmentation networks by weak supervision. To allow the segmentation network to learn more information under limited annotation data, we use both unlabeled and image-level labeled data to train the student-SN in the form of pseudo-labels and class activation maps. The uniqueness of this method lies in mining the potential benefits of classification to segmentation from two aspects to alleviate the problem of the small amount of pixel-level labeled data. On the one hand, we employ quality-CN to evaluate the quality level of pseudo-labels and provide reliable pseudo-labels to student-SN for self-training. On the other hand, we use disease-CN to generate accurate CAMs to transfer the localization prior to student-SN. We have introduced the generation method of reliable pseudo-labels in Section 3.2. Next, we will focus on the production and employment of CAMs.

#### 3.3.1. Generating CAMs

CAM is first proposed by [7] through global average pooling. A CAM for a particular category indicates the discriminative image regions used by CNN to identify that category. The CAM approach can localize objects from a classification model [38], which is widely used in weakly supervised semantic segmentation. However, in most circumstances, the CAMs directly generated by the classification network are not precise enough. The masks generated by the segmentation network possess the location and contour information of the skin lesion. Therefore, we employ masks to promote the disease-CN to generate precise CAMs.

We use the classification training dataset $D_{l}^{I}$ and the masks generated by the teacher-SN to train the skin disease classification network disease-CN. Each classification training image and its corresponding lesion mask are concatenated as an input to disease-CN, which aims to enhance disease-CN’s location ability to produce accurate CAMs.
**Algorithm** **1:** Generate reliable pseudo-labels  **Input:** Pixel-level labeled dataset $D_{l}^{P}={\left\{\left(X_{l}^{P},Y_{l}^{P}\right)\right\}}_{i=1}^{N}$, unlabeled dataset $\;\;\;\;\;\;\;\;\;\;\;\;\;\;\;\;\;D_{u}={\left\{X_{u}\right\}}_{i=1}^{n}$, teacher-SN *T*, quality-CN *Q*
  **Output:** Reliable pseudo-labels and corresponding images
  _**1**_  // Train *T* to generate pseudo-labels
  _**2**_  Train *T* on $D_{l}^{P}$ and save *k* checkpoints ${\left\{T_{j}\right\}}_{j=1}^{k}$
  _**3**_  // Train *Q* to screen reliable pseudo-labels
  _**4**_  for $X_{l}^{P}\in D_{l}^{P}$ do
  _**5**_           for $T_{j}\in {\left\{T_{j}\right\}}_{j=1}^{k}$ do
  _**6**_                    Generate pseudo-label $Y_{pseudo}^{P^{\prime }}=T_{j}(X_{l}^{P})$
  _**7**_                    Compute the Jaccard score *s* with Equation (1) between $Y_{l}^{P}$ and $Y_{pseudo}^{P^{\prime }}$
  _**8**_                    The category $Y_{pseudo}^{I}$ of $Y_{pseudo}^{P^{\prime }}$ is set according to *s* by Equation (2)
  _**9**_  Denote the pseudo-label quality level training set as $D_{pseudo}^{I}$.          $D_{pseudo}^{I}={\left\{\left(X_{l}^{P},Y_{pseudo}^{P^{\prime }}\right),Y_{pseudo}^{I}\right\}}_{i=1}^{kN}$  _**10**_  Train Q on $D_{pseudo}^{I}$
 _**11**_  // Obtain reliable pseudo-labels from $D_{u}$
 _**12**_  $D_{pseudo}^{P}=\{\}$
 _**13**_  $n^{\prime }=0$
 _**14**_  for $X_{u}\in D_{u}$ do
 _**15**_           Generate pseudo-label $T(X_{u})$
 _**16**_           If $Q(X_{u},T(X_{u}))=1$
 _**17**_                    $D_{pseudo}^{P}=D_{pseudo}^{P}\cup (X_{u},T(X_{u}))$
 _**18**_                    $n^{\prime }++$
 _**19**_  $D_{pseudo}^{P}={\left\{X_{u},T(X_{u})\right\}}_{i=1}^{n^{\prime }}$
 _**20**_  **Return**
$D_{pseudo}^{P}$


#### 3.3.2. Refine Segmentation

**Figure 4:**
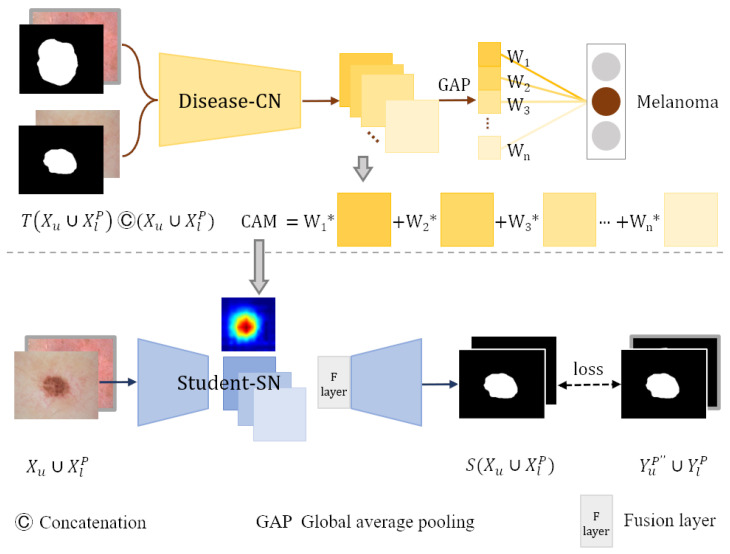
Student-SN learning in weakly labeled data.

As shown in Figure 4, images from segmentation training dataset $\left(D_{l}^{P}\cup D_{pseudo}^{P}\right)$ and corresponding masks are concatenated along the dimension of the channels and then input into the trained disease-CN. We weight the feature maps produced by the last convolutional layer of disease-CN using the class-specific weights of the output layer. Then, all channels of the weighted feature maps are summed to generate the CAMs.

The backbone network of the student segmentation network student-SN is the same as that of teacher-SN. To migrate the lesion location information from the CAMs into the student-SN, we add a fusion layer after the encoder of the student-SN. The feature maps extracted by the encoder are stitched with the CAMs along the channel dimension. The fusion layer fuses the spliced information using a convolutional layer with post-conjugated BN and ReLU activation functions. Then, the fused feature maps are fed into the decoder to refine segmentation. The enhanced CAMs are employed as a prior to help the student-SN learn the location information of lesions and reduce the need for dense pixel-level annotations. In addition, the student-SN is trained with the pixel-level labeled dataset $D_{l}^{P}$ and pseudo-label dataset $D_{pseudo}^{P}$ to learn more about image features.

### 3.4. Utilizing Masks to Classify

In clinical environments, pathologists generally diagnose melanoma according to the lesion border information. Pigmented nevi are generally symmetrical in shape, mostly round, with well-defined margins. Melanoma is asymmetrical in shape, with irregular and indistinct margins. The contour information of the lesion is crucial for the diagnosis of melanoma. In addition, noise in the dermoscopic image (such as hairs and bubbles) may interfere with the discrimination of disease-CN. With the assistance of quality-CN and disease-CN, the student-SN’s segmentation performance may be improved by obtaining information about the image feature and lesion position from pseudo-labels and CAMs. Therefore, we employ the segmentation masks generated by the student-SN to provide contour information and help the disease-CN focus on areas of skin lesions that are more meaningful for diagnosis, reducing the impact of noise and relatively unimportant background areas on category determination.

The disease-CN’s structure is roughly the same as quality-CN. The difference is that after performing global average pooling, the features are input to a randomly initialized fully connected layer with three neurons followed by a softmax activation function. We use the skin disease classification training set $D_{l}^{I}$ to train the disease-CN. During the training phase, the images $X_{l}^{I}$ and masks $T\left(X_{l}^{I}\right)$ are concatenated along the channel dimension as the input to the disease-CN, aiming to improve the diagnostic performance of disease-CN for skin disease.

## 4. Experiments

### 4.1. Dataset

We evaluate the proposed CL-DCNN model on two dermoscopic image datasets.

(1) ISIC 2017: ISIC 2017 is a skin lesion segmentation and classification dataset provided by the International Skin Imaging Collaboration Organization. The ISIC 2017 dataset included 2000 dermoscopic images for training, 150 for validation, and 600 for testing. Each dermoscopic image has its corresponding pixel-level expert annotation for segmentation and a gold-standard diagnosis of lesions (melanoma, mole, and seborrheic keratosis) for classification. We use the pixel-level labeled data of ISIC 2017 to train the teacher-SN and student-SN to segment the skin lesions, and we use the image-level labeled data to train the disease-CN to diagnose the type of skin disease.
(2) ISIC Archive: ISIC Archive is a skin lesion classification dataset that contains 1320 image-level annotated dermoscopic images. It comprises 466 cases diagnosed with melanoma, 32 cases with seborrheic keratosis, and 822 cases with nevus. We use ISIC Archive to expand the disease-CN’s training data and serve the ISIC Archive as unlabeled data to generate pseudo-labels. The relevant information of the dataset is shown in Table 2.

### 4.2. Evaluation Metrics

(1) Segmentation evaluation metrics: we use five indicators of the Jaccard index (JA), Dice coefficient (DI), pixel-wise accuracy (pixel-AC), pixel-wise sensitivity (pixel-SE), and pixel-wise specificity (pixel-SP) to evaluate the segmentation performance.
(2) Classification evaluation metrics: we use four indicators of the area under receive operation curve (AUC), accuracy (AC), sensitivity (SE), and specificity (SP) to evaluate the classification performance.

### 4.3. Experimental Details

The teacher-SN is trained on the pixel-level labeled dataset ISIC 2017. We input the images from ISIC 2017 into *k* (Empirically set to 5) different checkpoints of the trained teacher-SN to generate pseudo-labels. Based on the similarity between the pseudo-labels and ground truth from ISIC 2017, a classification training set $D_{pseudo}^{I}$ presents the quality level of the pseudo-labels is generated, and the quality-CN is trained on $D_{pseudo}^{I}$. The images without pixel-level annotations from ISIC Archive are input into the trained teacher-SN to generate pseudo-labels. The quality of the pseudo-labels are evaluated by quality-CN to obtain a reliable pixel-level pseudo-label training set $D_{pseudo}^{P}$. The disease-CN is trained on image-level labeled datasets ISIC 2017 and ISIC Archive. The student-SN is trained on pixel-level labeled datasets ISIC 2017 and $D_{pseudo}^{P}$.

Before training the network, the images are preprocessed by the random affine transformation, vertical flip, horizontal flip, and other data enhancement operations to increase the data’s diversity and prevent overfitting. We use the adam algorithm to optimize the networks. The initial learning rates are set to 0.0001, and the maximum iteration period is 500. The ISIC 2017 validation set is used to monitor the CL-DCNN model’s convergence and terminates the training process if the model falls into overfitting. In the testing phase, the trained CL-DCNN model is directly applied to the ISIC 2017 testing set to evaluate the skin lesion segmentation and classification performance.

### 4.4. Experimental Results

#### 4.4.1. Segmentation Results

**Figure 5:**
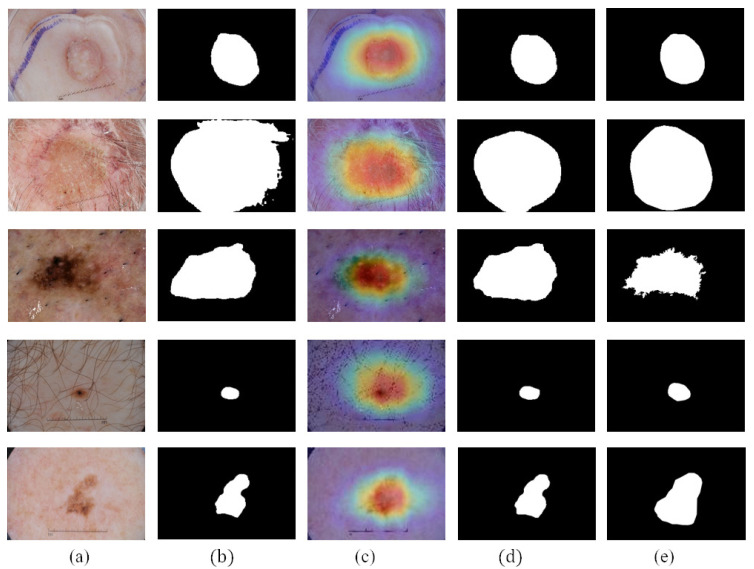
Segmentation results generated by the CL-DCNN model at each stage: (**a**) dermoscopy images, (**b**) segmentation results generated by teacher-SN, (**c**) CAMs generated by disease-CN, (**d**) segmentation results generated by student-SN, and (**e**) ground truth.

We compare the segmentation performance of the CL-DCNN model with other skin disease segmentation methods on the ISIC 2017 testing set. These segmentation methods include FCN [39], U-Net [40], generative adversarial networks with dual discriminators DAGAN [13], edge and neighborhood guidance network ENGNet [41], neighborhood context refinement network NCRNet [42], AG-Net [43] and MultiResUNet [44]. It can be seen from Table 3 that the CL-DCNN has achieved superior segmentation results in the three indicators of JA, DI, and Pixel-AC. The CL-DCNN model achieved a JA of 79.1, DI of 86.7, and Pixel-AC of 94.1. Specifically, our model achieves a 79.1 JA, which is 0.5% higher than the second-best model NCRNet’s. To demonstrate the performances of our proposed method, we visualize the segmentation results at each stage of the CL-DCNN model in Figure 5. The CL-DCNN model achieves more accurate segmentation results in the second stage, which is closer to the ground truth.

#### 4.4.2. Classification Results

Table 4 shows the average classification performance of the CL-DCNN model. We compare it with several classification methods: Xception [36], advanced semi-supervised adversarial classification model SSAC [45], attention residual learning convolutional neural network ARL-CNN [19], synergic deep learning model SDL [46], MWNL-CLS [47], and mutual bootstrapping deep convolutional neural networks MBDCNN [23]. The CL-DCNN model can obtain the highest AC, SP, and AUC compared to other approaches. The CL-DCNN model achieved an AUC of 93.7, SP of 94.7, and AC of 90.7, which improves the AUC by 0.9%, SP by 0.4%, and AC by 0.1%. The substantial performance gains over the base model and five recent solutions indicate the superiority of the proposed CL-DCNN model.

#### 4.4.3. Advantages of CAMs and Pseudo-Labels

The uniqueness of the proposed skin lesion self-training segmentation method lies in that the student-SN can learn from both CAMs and pseudo-labels (PLs). We transfer the high-quality lesion area activation maps to the student-SN to improve its localization ability. Moreover, we provide a reliable pseudo-label generate method based on the similarity between pseudo-labels and ground truth for selective retraining.

**Figure 6:**
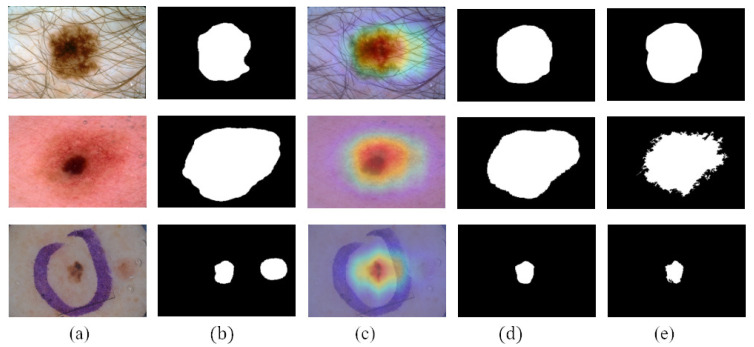
Comparison of the segmentation results obtained by segmentation network with or without using the CAMs: (**a**) dermoscopy images, (**b**) segmentation results obtained when not using CAMs, (**c**) CAMs, (**d**) segmentation results obtained when using CAMs, and (**e**) ground truth.

CAMs can build a generic localizable deep representation that exposes the implicit attention of CNNs on an image [7]. To exhibit the effectiveness of CAMs, we visualize the segmentation results with and without CAMs in Figure 6. Figure 6c shows that CAMs can activate the lesion area—the closer to the lesion center, the higher the network response. In addition, the location of CAMs is close to the ground truth. Therefore, we use CAMs to assist the segmentation network to obtain the information of the lesion location. As shown in Figure 6d, with the help of CAMs, the segmentation results generated by our work are more consistent with ground truth than those not using CAMs. Consequently, we believe that CAMs could help promote CL-DCNN to better locate the lesion area to achieve better segmentation performance.

**Figure 7:**
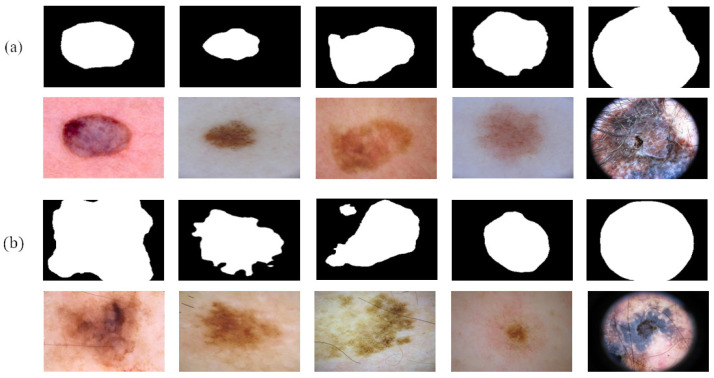
Comparison between reliable pseudo-labels and unreliable pseudo-labels: (**a**) reliable pseudo-labels, corresponding images, and (**b**) unreliable pseudo-labels, corresponding images.

Figure 7 shows the reliable pseudo-labels and unreliable pseudo-labels evaluated by quality-CN. The contour of the reliable pseudo-labels is consistent with the area of skin lesions. The screened reliable pseudo-labels show the potential to help the student-SN to reduce the need for pixel-level labeled data and learn from image features.

To verify the validity of the CAMs and pseudo-labels, we implement the following ablation experiments, as shown in Table 5. The results show that the segmentation performance is improved, including JA, DI, and Pixel-SE, after using CAMs and reliable pseudo-labels. Comparing to the base model, our model improves the average JA by 0.8%, DI by 0.7%, and Pixel-SE by 2.6%. Significantly, the JA improved from 78.3% to 79.1%. These results prove that the CAMs and pseudo-labels have the ability to improve segmentation performance.

#### 4.4.4. Advantages of Masks

**Figure 8:**
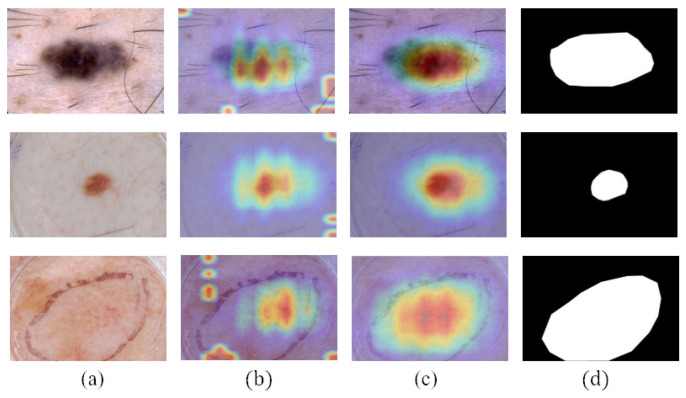
Comparison of the CAMs obtained by disease-CN with or without using the lesion masks: (**a**) dermoscopy images, (**b**) CAMs obtained when not using lesion masks, (**c**) CAMs obtained when using lesion masks, and (**d**) ground truth.

In order to represent the impact of masks on the performance of classification network, we visualized the network’s attention in the form of CAMs in Figure 8. CAMs could visualize the predicted class scores on a given image, highlighting the discriminative object parts detected by the CNN. It can be seen that after using the masks generated by teacher-SN, disease-CN will pay more attention to the lesion area. The information on lesion area plays an essential role in skin disease judgment.

Table 6 shows the average classification performance of melanoma and seborrheic keratosis when nothing migrated to the disease-CN, and the masks generated by the teacher-SN and student-SN migrated to the disease-CN. After using the masks generated by teacher-SN, the classification performance improved by 0.2 of AC, 1.2 of SP, and 0.7 of AUC. Significantly, after using the masks generated by student-SN, the classification performance improved by 0.9 of AC, 0.7 of SE, and 0.9 of AUC. It can be seen that masks are useful in improving classification performance. The masks generated by the student-SN can assist the disease-CN in obtaining better classification performance. We conduct that masks provide the location and contour information of skin lesions, in which discriminative features would be extracted by disease-CN. The higher the accuracy of the masks, the more it can promote the classification network to achieve accurate classification performance.

## 5. Conclusions

In this paper, we proposed a CL-DCNN model for the collaborative learning of dermatological segmentation and classification. The model fully exploits the correlation between tasks under limited annotation data, allowing the segmentation and classification network to learn more information. The experimental results show that the skin lesion segmentation performance can be improved by using the reliable pseudo-labels screened by the classification network and target location maps generated by the classification network. In addition, the accurate masks produced by the segmentation network help improve the discriminative ability of classification. The limitations of this method are mainly reflected in the generalization of the model, which is challenging to apply to clinical practice. We have tried applying the model to other data, but the result is not ideal. Therefore, a potential work is to explore how to make the prediction results of other modality data accurate. In the future, we plan to extend this framework to domain adaptation to improve the model’s generalization ability.

## Figures and Tables

**Table 1:** Terms and corresponding definitions.

Terms	Definitions
Teacher-SN	Teacher-SN is a teacher segmentation network used to generate pseudo-labels and masks.
Disease-CN	Disease-CN is a disease classification network used to diagnose skin disease types.
Quality-CN	Quality-CN is a pseudo-label quality evaluation network for screening reliable pseudo-labels.
Student-SN	Student-SN is a student segmentation network for the fine segmentation of skin lesion regions.
$D_{l}^{P}={\left\{\left(X_{l}^{P},Y_{l}^{P}\right)\right\}}_{i=1}^{N}$	$D_{l}^{P}$ is a segmentation training set that contains *N* dermoscopy images ${\left\{X_{l}^{P}\right\}}_{i=1}^{N}$ and corresponding pixel-level labels ${\left\{Y_{l}^{P}\right\}}_{i=1}^{N}$. Some pixels in $Y_{l}^{P}$ belong to the skin lesion area, and others belong to the normal skin area.
$D_{l}^{I}={\left\{\left(X_{l}^{I},Y_{l}^{I}\right)\right\}}_{i=1}^{M}$	$D_{l}^{I}$ is a classification training set that contains *M* dermoscopy images ${\left\{X_{l}^{I}\right\}}_{i=1}^{M}$ and corresponding image-level labels ${\left\{Y_{l}^{I}\right\}}_{i=1}^{M}$. $Y_{l}^{I}$ represents the type of skin disease (melanoma, nevus, or seborrheic keratosis).
$D_{u}={\left\{X_{u}\right\}}_{i=1}^{n}$	$D_{u}$ is an unlabeled segmentation training set that contains *n* dermoscopy images ${\left\{X_{u}\right\}}_{i=1}^{n}$.

**Table 2:** Details of dataset ISIC 2017 and ISIC Archive.

Dataset	Format	Label	Train	Size Validation	Testing
ISIC 2017	Png	Pixel-level	2000	150	600
		Image-level			
ISIC Archive		Image-level	1320	0	0

**Table 3:** Segmentation performance of the CL-DCNN model and other skin lesion segmentation methods on the ISIC 2017 testing set. The highest results are shown in bold font for easy observation and analysis.

Method	JA	DI	Pixel-AC	Pixel-SE	Pixel-SP
FCN [39]	75.2	84.1	93.9	82.2	97.0
U-Net [40]	76.5	85.2	93.3	84.5	97.3
DAGAN [13]	77.1	85.9	93.5	83.5	97.6
ENGNet [41]	77.1	85.3	93.2	82.7	**97.8**
NCRNet [42]	78.6	86.6	94.0	**86.9**	95.9
AG-Net [43]	76.9	85.3	93.5	83.5	97.4
MultiResUNet [44]	76.8	85.2	93.6	83.9	96.8
Ours	**79.1**	**86.7**	**94.1**	86.5	95.9

**Table 4:** Comparison of the average classification performance between CL-DCNN model and other skin lesion classification methods on the ISIC 2017 testing set. The highest results are shown in bold font for easy observation and analysis.

Method	AC	SE	SP	AUC
Xception [36]	89.8	70.1	94.3	92.8
ARL-CNN [19]	86.4	76.3	88.2	91.7
SSAC [45]	86.2	73.6	91.0	91.6
SDL [46]	90.6	-	-	91.3
MWNL-CLS [47]	76.3	56.4	76.0	91.7
MBDCNN [23]	90.4	**78.5**	93.0	-
Ours	**90.7**	70.8	**94.7**	**93.7**

**Table 5:** Segmentation performance of the CL-DCNN model on the ISIC 2017 testing set after training with CAMs and pseudo-labels. The highest results are shown in bold font for easy observation and analysis.

Methods		JA	DI	Pixel-AC	Pixel-SE	Pixel-SP
CAMs	PLs					
		78.3	86.0	94.1	83.9	**97.7**
✓		78.9	86.5	**94.3**	85.0	96.7
✓	✓	**79.1**	**86.7**	94.1	**86.5**	95.9

**Table 6:** Comparison of average classification performance with or without masks. The highest results are shown in bold font for easy observation and analysis.

Methods		AC	SE	SP	AUC
Teacher-SN’s Mask	Student-SN’s Mask				
		89.8	70.1	94.3	92.8
✓		90.0	65.2	**95.5**	93.5
	✓	**90.7**	**70.8**	94.7	**93.7**

## Data Availability

The data used to support the findings of this study are available from the corresponding author upon request.
